# Sarcopenia Risk is Associated With Decreased Swallow Safety in Patients With Dysphagia

**DOI:** 10.1002/ohn.70172

**Published:** 2026-02-18

**Authors:** Nogah Nativ‐Zeltzer, Yarden Ashkenazi, Lisa S. Halaby, Liav Hayat, Oshri Wasserzug, Yael Oestreicher‐Kedem, Nidal Muhanna, Yuval Nachalon

**Affiliations:** ^1^ Department of Communication Disorders, Gray Faculty of Medical & Health Sciences Tel Aviv University Tel Aviv Israel; ^2^ Department of Otolaryngology Head and Neck Surgery and Maxillofacial Surgery, Tel‐Aviv Sourasky Medical Center Tel‐Aviv Israel; ^3^ School of Medicine Department of Otolaryngology, Head and Neck Surgery, Gray Faculty of Medical & Health Sciences, Tel Aviv University Tel‐Aviv Israel

**Keywords:** aging, dysphagia, handgrip strength, sarcopenia, swallowing function

## Abstract

**Objective:**

Sarcopenia, characterized by the loss of skeletal muscle mass and strength, is associated with adverse health outcomes in older adults. This study aimed to evaluate the relationship between sarcopenia risk, handgrip strength, and swallowing function in patients with dysphagia undergoing Fiberoptic Endoscopic Evaluation of Swallowing (FEES).

**Study Design:**

Retrospective chart review.

**Setting:**

A tertiary dysphagia clinic.

**Methods:**

Medical records of individuals aged 65 years and older who attended the dysphagia clinic over a 6‐month period were reviewed. Data collected included demographics, clinical symptoms, handgrip strength, SARC‐F (Strength, Assistance in walking, Rise from a chair, Climb stairs, and Falls) scores, Penetration Aspiration Scale (PAS) scores, and Yale residue scale scores from FEES. Critically weak grip strength was defined using previously established thresholds. Correlations between sarcopenia indicators and swallowing outcomes were analyzed using Spearman's rho.

**Results:**

Thirty‐three patients were included (mean age 77.6 ± 7.5 years; 52% male). Twenty‐five (76%) demonstrated critically weak grip strength, and 9 (27%) had SARC‐F ≥ 4, indicating sarcopenia risk. SARC‐F scores were negatively correlated with Functional Oral Intake Scale (FOIS) (rs = –0.45, *P* = .01) and positively correlated with liquid PAS (rs = 0.41, *P* = .02). Handgrip strength was negatively correlated with age (rs = –0.39, *P* = .03).

**Conclusions:**

Most older adults undergoing FEES exhibited critically weak grip strength, suggesting high sarcopenia risk. Higher sarcopenia risk correlated with worse swallowing safety and reduced oral intake. These findings highlight the importance of assessing and addressing nutritional and muscle strength deficits in dysphagic older adults.

**Level of Evidence:**

4.

Sarcopenia, a syndrome characterized by the progressive loss of skeletal muscle mass and strength, has been increasingly recognized as a critical factor of adverse health outcomes in older adults.^1^ The condition is associated with a heightened risk of falls, fractures, functional decline, and mortality.^2^, ^3^ Sarcopenia can result from muscle fiber changes, particularly the reduction of the number and size of type II (fast‐twitch) fibers, decreased muscle protein synthesis, and hormonal changes which diminish muscle growth and repair.^4^, ^5^, ^6^ Oxidative stress may also contribute by causing damage to the mitochondria, leading to increased production of reactive oxygen species, which promote muscle degradation.^7^ Physical inactivity and nutritional deficits also contribute to the development of sarcopenia.^8^, ^9^

Recent studies have suggested that sarcopenia may be associated with dysphagia or impaired swallowing function among the elderly.^10^, ^11^, ^12^, ^13^ Generalized sarcopenia may also lead to the atrophy of muscles involved in swallowing, reducing their ability to generate adequate force for effective swallowing and maintain deglutitive airway protection. Sarcopenia can also affect the muscles involved in respiration, such as the diaphragm and intercostal muscles. Weakness in these muscles can impair the coordination between breathing and swallowing, which is critical for preventing aspiration.^14^, ^15^, ^16^ The intersection of sarcopenia and dysphagia presents a significant clinical concern, as impaired swallowing can exacerbate malnutrition and frailty, creating a vicious cycle that further deteriorates muscle strength and function.

The relationship between screening tests for sarcopenia and Fiberoptic Endoscopic Evaluation of Swallowing (FEES)‐based swallowing outcomes is not well established. This study aimed to investigate the correlation between sarcopenia risk, handgrip strength, and swallowing dysfunction in patients aged 65 years and older undergoing FEES.

## Materials and Methods

This retrospective cohort study was conducted at a tertiary referral center's dysphagia clinic over a span of 8 months. The study protocol was approved by the Tel Aviv Medical Center institutional review board. Inclusion criteria were: (1) age ≥65 years, (2) referral for dysphagia evaluation, (3) completion of FEES examination, and (4) available handgrip strength measurements. Exclusion criteria included: (1) acute neurological conditions within 3 months, (2) active cancer treatment, (3) inability to complete handgrip testing, and (4) incomplete swallowing assessment data.

The data collected comprised of patient demographics, clinical symptoms, functional oral intake scale (FOIS), a 7‐point ordinal scale developed to document the functional level of oral intake of food and liquid in patients with dysphagia.^17^ as well as the EAT‐10 questionnaire, EAT‐10 (Eating Assessment Tool‐10) is a self‐administered, symptom‐specific questionnaire for dysphagia.^18^ Additional data included handgrip strength measurements, SARC‐F (Strength, Assistance in walking, Rise from a chair, Climb stairs, and Falls) score,^19^ Penetration Aspiration Scale (PAS) Score^20^ and the Yale residue scale score from FEES.^21^ Critically weak grip was defined based on previously established normative data and risk thresholds of handgrip strength.^22^ Details on the procedures are described below.

### SARC‐F (Strength, Assistance in Walking, Rise From a Chair, Climb Stairs, and Falls) Score

The SARC‐F is a 5‐item questionnaire that asks about difficulties in strength, walking, getting up from a chair, climbing stairs, and history of falls. A score ≥4 points on the SARC‐F is suggestive of sarcopenia.^19^

### Hand Grip Strength Measurement

**Figure 1:**
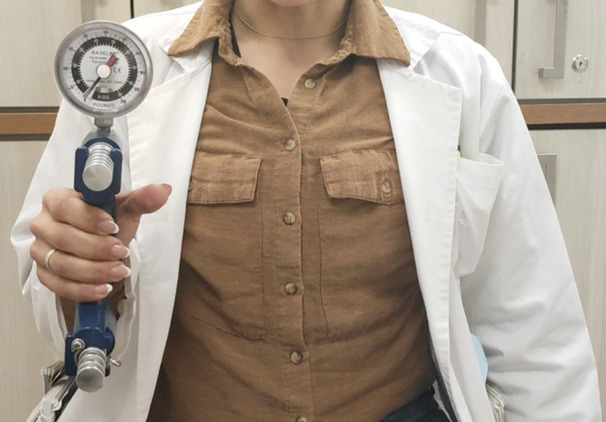
Handgrip strength was measured using a Baseline Hydraulic Hand Dynamometer, with participants seated, shoulder adducted, elbow flexed at 90°, and forearm in neutral.

Handgrip strength is widely used as a simple, reliable indicator of overall muscle strength and has been correlated with sarcopenia. Critically weak handgrip strength has been identified as a predictor of mortality and poor health outcomes in older adults.^23^, ^24^, ^25^ Handgrip strength in kilograms was measured with a Baseline Hydraulic Hand Dynamometers (Fabrication Enterprises Inc.). Participants were seated with their shoulder adducted, elbow flexed at 90°, and forearm in neutral position (Figure 1). Three maximum voluntary contractions were performed with the dominant hand, with 30‐second rest periods between attempts. The highest value was recorded for analysis. Critically weak grip strength was defined using age‐, sex‐, and height‐specific cutoff values.^22^

### FEES Assessment

The FEES assessment was conducted using a video laryngoscope (VNL11‐J10; PENTAX Europe GmbH). The laryngoscope was inserted through the nose without the application of a topical anesthetic or vasoconstrictor to the nasal mucosa and positioned in either the nasopharynx or oropharynx. Participants then underwent various swallowing tasks, including the following protocol: swallows of colored water (International Dysphagia Diet Standardization Initiative (IDDSI) 0) in specific volumes (two 5 ml, two 10 ml, 2 free sips from a cup, and continuous sips of 3‐4 swallows through a straw), followed by applesauce (IDDSI 4; 2 spoons of 5 ml) and bites of a biscuit (IDDSI 7; 2 bites). After each swallow, the pharynx was examined for residue in the vallecula and the pyriform sinus, with grading performed according to the Yale Pharyngeal Residue Severity Rating Scale.^21^ If residue was present, participants were prompted to swallow again to clear it, with additional swallows provided if strategies were employed. Aspiration and penetration were assessed for each swallow and graded using the PAS score.^20^ The assessments were conducted by a trained laryngologist and speech‐language pathologist.

### Statistical Analysis

Statistical analysis was conducted using SPSS version 29.0 (IBM Inc.). Correlation between ordinal metrics was assessed using Spearman's rho correlation coefficient and correlations between continuous measures was assessed using Pearson correlation coefficient. A one‐way analysis of variance (ANOVA) with Bonferroni correction for multiple comparisons was used to compare the groups of participants with critically weak hand grip to those with normal hand grip. A *P* value of .05 was set as a criterion for statistical significance.

## Results

### Patient Characteristics

Medical records of 85 individuals who attended the dysphagia clinic were reviewed. Forty patients were aged 65 years or older. Of these, 3 did not complete handgrip strength measurements, 1 presented with an acute stroke, and 3 were undergoing active cancer treatment at the time of evaluation and were therefore excluded. The remaining 33 patients were included in the final analysis. The mean age ±SD was 77.6 ± 7.5 years, with 17 (52%) being male. Solid food dysphagia was reported in 8 (24%) individuals and 16 (48%) experienced both liquid and solids dysphagia. Weight loss was reported in 5 (15%) individuals and 5 (15%) experienced pneumonia in the 6 months prior to their clinic visit. Table 1 presents the demographic and clinical characteristics of the study population.

**Table 1:** Clinical Characteristics of Study Population (N = 33)

Characteristic	Value
Age (years), mean ± SD	77.6 ± 7.5
EAT‐10, mean ± SD	13.6 ± 10.6
FOIS, mean ± SD	6.3 ± 1.2
Comorbidities, n (%)	
Neurodegenerative disease	4 (12)
Chronic stroke (>3 months)	3 (9)
Epilepsy/Schizophrenia	2 (6)
Head & neck malignancy	6 (18)
Zenker's diverticulum/Globus	2 (6)
Spine surgery history	1 (3)
GERD/hiatal hernia	6 (18)
COPD/asthma	4 (12)
No dysphagia‐related comorbidities reported	8 (24)

Comorbidities were not mutually exclusive (patients could be included in more than one category). Percentages are calculated out of the total cohort (N = 33) and rounded to the nearest whole number.

Abbreviations: COPD, chronic obstructive pulmonary disease; CVA, cerebrovascular accident; EAT‐10, Eating Assessment Tool‐10; FOIS, Functional Oral Intake Scale; GERD, gastroesophageal reflux disease; SD, standard deviation.

### Swallowing Function Assessment

Mean FOIS score was 6.3 ± 1.2 and the mean EAT‐10 score was 13.6 ± 10.6. To provide clinical context for the swallowing assessments used in this study, we briefly outline commonly used clinical reference points for the Functional Oral Intake Scale (FOIS) and the Eating Assessment Tool‐10 (EAT‐10). The FOIS is a validated 7‐point ordinal scale designed to document the functional level of food and liquid intake, where a score of 7 represents a normal, unrestricted diet and a score of 1 indicates no oral intake.^17^ Mean FOIS score was 6.3 ± 1.2, indicating that most participants were consuming an oral diet with some degree of limitation or required compensatory strategies. Subjective dysphagia severity was further quantified using the EAT‐10, a 10‐item survey where a total score greater than 3 is clinically defined as abnormal.^18^ The mean EAT‐10 score was 13.6 ± 10.6, exceeding the established abnormal threshold (>3), but remaining just below levels commonly associated with high aspiration risk (>15).^26^ Together, these findings suggest a cohort with clinically meaningful, yet predominantly mild‐to‐moderate swallowing dysfunction. Additional outcomes of the swallowing assessment are detailed in Table 2.

**Table 2:** FEES and Sarcopenia‐Related Measurements

FEES measurements	Mean ± SD
Measurements	
Liquid	
PAS	1.9 ± 1.9
Yale Vallecula	1.5 ± 0.6
Yale Pyriform Sinuses	1.4 ± 0.7
Soft food	
Yale Vallecula	1.6 ± 0.8
Yale Pyriform Sinuses	1.2 ± 0.4
Solid Food	
Yale Vallecula	1.5 ± 0.7
Yale Pyriform Sinuses	1.1 ± 0.3
Sarcopenia measures	
SARC‐F Score	2.3 ± 1.8
Hand grip strength (kg)	21.9 ± 7.8

Abbreviations: FEES, Fiberoptic Endoscopic Evaluation of Swallowing; PAS, Penetration Aspiration Scale; SARC‐F, Strength, Assistance in walking, Rise from a chair, Climb stairs, and Falls questionnaire.

### Sarcopenia Measures

Mean hand grip strength was 21.88 (±7.81) kg. Twenty‐five participants (76%) had critically weak grip strength based on age‐ and sex‐specific normative values. The mean SARC‐F scores were 2.28 (±1.81) and 9 participants (27%) had SARC‐F scores ≥4, indicating risk for sarcopenia.

### Relationship Between Sarcopenia Risk and Swallowing Measures

**Figure 2:**
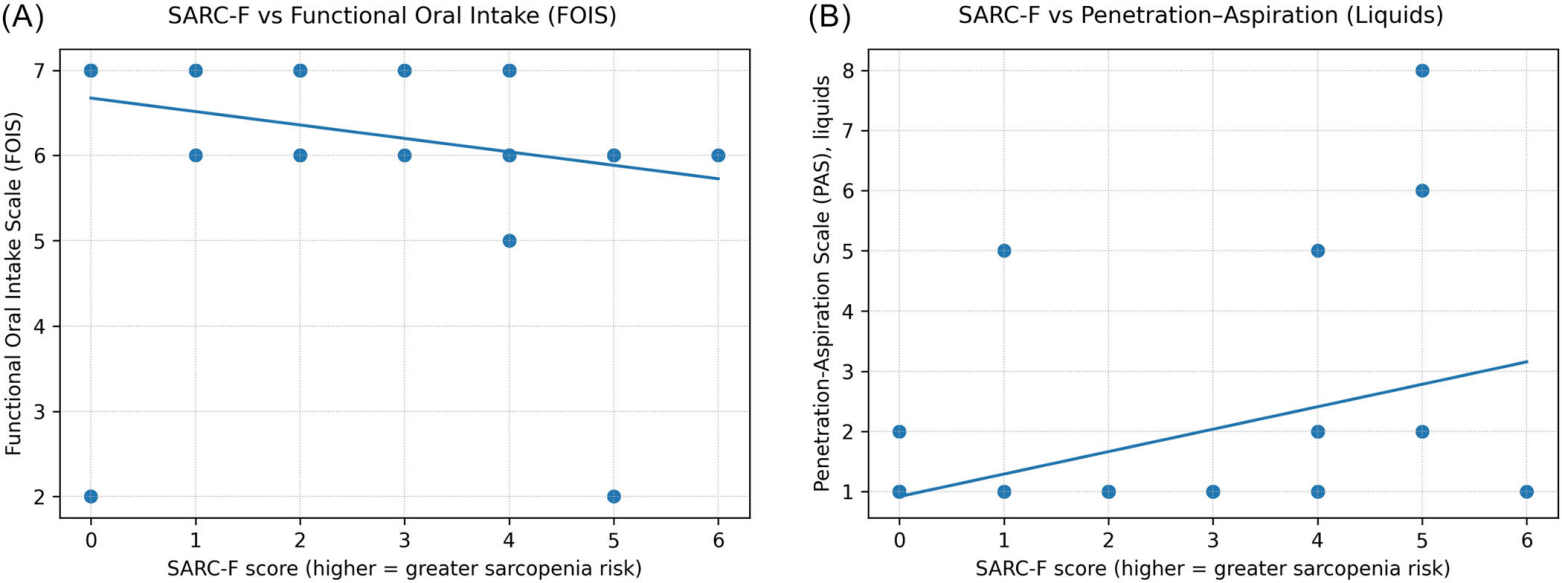
Association between sarcopenia risk and swallowing measures. (A) Relationship between SARC‐F score and Functional Oral Intake Scale (FOIS), demonstrating a moderate negative correlation (Spearman's *r*_s_ = −0.45, *P* = .01). (B) Relationship between SARC‐F score and Penetration–Aspiration Scale (PAS) scores for liquid swallows during FEES, demonstrating a moderate positive correlation (Spearman's *r*_s_ = 0.41, *P* = .02). Scatterplots are shown with linear trend lines for visualization.

SARC‐F scores showed a moderate negative correlation with FOIS (rs = −0.45, 95%, *P* = .01) and a moderate positive correlation with liquid PAS scores (rs = 0.41, 95% *P* = .02) (Figure 2). Hand grip strength demonstrated a moderate negative correlation with age (rs = −0.39, *P* = .03) but did not significantly correlate with swallowing measures. No significant differences in swallowing measures were observed between patients with critically weak versus normal hand grip strength (*P* > .05).

## Discussion

This study evaluated the relationship between sarcopenia risk, hand grip strength, and swallowing dysfunction in elderly patients undergoing FEES, identifying 2 key findings. First, 76% of patients exhibited critically weak grip strength, a prevalence significantly higher than that reported in the general population: 5% to 13% in individuals aged 60 to 70 and 11% to 50% in those aged 80 or older.^26^, ^27^, ^28^, ^29^, ^30^ Second, patient‐reported sarcopenia risk (SARC‐F) was significantly associated with swallowing safety and functional oral intake.

The association between SARC‐F scores and both functional oral intake and aspiration risk in this cohort highlights that dysphagia and sarcopenia may share common pathophysiological mechanisms. Generalized muscle weakness, a hallmark of sarcopenia, can involve muscles critical for functional swallowing. Feng et al reported an association between geniohyoid muscle atrophy and aspiration in healthy older adults, highlighting the role of the geniohyoid in hyolaryngeal elevation and airway protection during swallowing.^31^ The relationship between sarcopenia and swallowing function is likely more pronounced in patients with dysphagia. Sporns et al found that muscle volume of key swallowing muscles, such as the digastric and geniohyoid, was inversely related to dysphagia severity in stroke patients.^10^ Similarly, decreased muscle mass was an independent risk factor for dysphagia in hospitalized patients.^32^ A recent study found that vocal fold atrophy in patients with Parkinson's Disease was predictive of reduced swallowing safety.^33^ These findings emphasize the importance of considering both general frailty and specific swallowing function when evaluating and treating elderly patients with dysphagia.

The bidirectional relationship between sarcopenia and dysphagia is evident in the findings of the current study. Greater sarcopenia risk was associated with worse functional oral intake status and increased airway invasion during liquid swallowing, as measured by PAS scores during FEES. This relationship is also supported in previous studies. In a cohort of 82 elderly hospitalized patients without initial dysphagia who had restricted oral intake for over 2 days, 26% of them developed dysphagia, all of whom also had sarcopenia.^32^ Since these patients did not have a medical etiology that could cause dysphagia, these findings suggest that low activity of swallowing muscles due to lack of oral intake in patients with sarcopenia may contribute to the development of dysphagia. Additionally, Bise et al demonstrated that improved nutritional status in post‐stroke patients was positively correlated with dysphagia recovery and inversely correlated with sarcopenia severity, highlighting the cyclical nature of these conditions.^34^ Collectively, these findings emphasize the need for integrated interventions addressing both nutritional status and muscle function in elderly patients with dysphagia, as reduced oral intake can initiate muscle atrophy critical for swallowing, while existing muscle weakness further compromises swallowing function.

While SARC‐F scores were associated with swallowing safety, hand grip strength did not directly correlate with swallowing outcomes in this study. This finding aligns with Butler et al who found no significant difference in hand grip strength between aspirators and nonaspirators.^35^ However, other studies have reported associations between hand grip strength and swallowing function, although they did not assess aspiration status directly.^36^, ^37^ These conflicting results may be attributed to variance in study populations, measurement techniques, and study design. Nevertheless, the high prevalence of critically weak handgrip strength (76%) observed in this dysphagic cohort compared to general population norms (5%‐13% for ages 60‐70), suggests a substantial burden of global vulnerability and frailty in this specific population. Handgrip strength did not directly correlate with swallowing safety or efficiency measures, indicating that it may not function as a swallowing‐specific severity marker in this clinical context. Rather, handgrip weakness may reflect broader systemic factors common in patients referred to tertiary dysphagia clinics, including multimorbidity, reduced physical activity, nutritional compromise, and overall functional decline. The clinical significance of this relationship is underscored by Steiber et al who found that individuals with critically weak hand grip strength had an 86% higher risk of mortality over 8 years compared to their age and height‐matched peers.^22^ Further studies are needed to clarify the relationship between hand grip strength and swallowing function, particularly given its established value as a predictor of overall health outcomes in elderly populations.

These findings suggest potential implications for clinical practice. The significant correlation between SARC‐F scores and swallowing safety indicators underscores the potential utility of this simple screening tool in identifying patients who may be at higher risk for aspiration. This tool may better capture the multidimensional functional decline relevant to swallowing safety than isolated peripheral strength measures. However, further research is needed to validate these findings in larger and more diverse cohorts before routine integration into clinical protocols.

This study has several limitations. The sample size was relatively small, potentially limiting the detection of additional statistically significant relationships. Furthermore, the data were collected from a single‐center tertiary care clinic, which may not represent other patient populations. Another limitation is the absence of direct muscle mass measurements, which could have provided a more comprehensive assessment of sarcopenia.

## Conclusions

This study highlights the significant prevalence of sarcopenia risk and critically weak hand grip strength among elderly patients with dysphagia. The associations between sarcopenia risk, impaired swallowing safety, and reduced oral intake suggest the need for integrated therapeutic approaches that address nutritional and muscle function deficits. Future research should focus on validating these findings in larger, more diverse cohorts and exploring the potential of targeted interventions to mitigate the cyclical relationship between sarcopenia and dysphagia.

## Author Contributions

**Nogah Nativ‐Zeltzer**, PhD, contributed substantially to the study design, data analysis, interpretation of results, and drafting of the manuscript; **Yarden Ashkenazi**, MA, assisted in data collection, data management, and manuscript preparation; **Lisa S. Halaby**, BA, assisted in data acquisition, data entry, and preliminary analyses; **Liav Hayat**, MA, contributed to data collection and organization of clinical data; **Oshri Wasserzug**, MD, provided clinical oversight, contributed to interpretation of findings, and critical manuscript revisions; **Yael Oestreicher‐Kedem**, MD, contributed to study conception, clinical evaluation, and critical revision of the manuscript; **Nidal Muhanna**, MD, PhD, supervised study design, provided methodological input, and contributed to interpretation and critical revision; **Yuval Nachalon**, MD, conceived the study, oversaw clinical and methodological aspects, and critically revised the final manuscript for important intellectual content.

## Disclosures

### Competing interests

None.

### Funding source

None.
